# Proceedings of the Central Ohio Dental Society

**Published:** 1876-02

**Authors:** M. DeCamp, H. J. Cressinger, L. W. Nevius


### PROCEEDINGS OF THE CENTRAL OHIO DENTAL SOCIETY.

This Society met persuant to adjournment, at Mansfield Ohio, in the dental rooms of L. W. Nevius.

In the absence of both President and Vice-President, T. S. Seely was called to act as President, pro tem.

Present—Drs. J. R. Cunningham, H. J. Cressinger, L. W. Nevius, J. A. Hisey, C. B. Mower, T. G. Bristor, T. S. Seely, C. R. Richards, Messrs. Holbrooks, H. Decamp and P. H. McNulty.

The following questions were discussed, in order, nearly all present participating.

1st. Dental Students.

2d. Irregularities.

3d. Dental Fees.

Several clinical operations were performed, after which the Society adjourned, to meet at Crestline, the 2d Monday in April. Dentists in the central part of the State, and all who can, are cordially invited to attend.

H. J. Cressinger, Sec'y.

M. DeCamp, Pres. L. W. Nevius, Cor. Sec'y.

